# Core-Shell Co/CoO Integrated on 3D Nitrogen Doped Reduced Graphene Oxide Aerogel as an Enhanced Electrocatalyst for the Oxygen Reduction Reaction

**DOI:** 10.3389/fchem.2016.00036

**Published:** 2016-08-22

**Authors:** Meng Wang, Yuyang Hou, Robert C. T. Slade, Jiazhao Wang, Dongqi Shi, David Wexler, Huakun Liu, Jun Chen

**Affiliations:** ^1^ARC Centre of Excellence for Electromaterials Science, Intelligent Polymer Research Institute, Australian Institute of Innovative Materials, University of WollongongWollongong, NSW, Australia; ^2^Department of Chemistry, University of SurreyGuildford, UK; ^3^Institute for Superconducting and Electronic Materials, Australian Institute of Innovative Materials, University of WollongongWollongong, NSW, Australia; ^4^School of Mechanical, Materials and Mechatronic Engineering, University of WollongongWollongong, NSW, Australia

**Keywords:** electrocatalyst, oxygen reduction reaction, Co/CoO, N-doped reduced graphene oxide aerogel (NGA), AEM fuel cell

## Abstract

Here, we demonstrate that Cobalt/cobalt oxide core-shell nanoparticles integrated on nitrogen-doped (N-doped) three-dimensional reduced graphene oxide aerogel-based architecture (Co/CoO-NGA) were synthesized through a facile hydrothermal method followed by annealing treatment. The unique endurable porous structure could provide sufficient mass transfer channels and ample active sites on Co/CoO-NGA to facilitate the catalytic reaction. The synthesized Co/CoO-NGA was explored as an electrocatalyst for the oxygen reduction reaction, showing comparable oxygen reduction performance with excellent methanol resistance and better durability compared with Pt/C.

## Introduction

Fuel cells (FCs) are believed to be promising energy conversion systems to satisfy today's increasing energy demands because of their high specific power density and low environmental impact (Liang et al., 2011). The cathodic oxygen reduction reaction (ORR) is considered to be the kinetically decisive step for the power conversion efficiency of FCs because of its sluggish reaction mechanism (Liang et al., 2011; Chen et al., 2014). As a commonly used electrocatalyst, platinum (Pt) is impeded from large-scale commercialization by its high cost, limited stability, and poor “poison” resistance (Liang et al., 2011; Liang J. et al., 2012; Chen et al., 2014). Therefore, numerous efforts have been devoted to developing novel electrocatalysts for the ORR with high efficiency, low cost, and environmental friendliness.

Recent studies have suggested that transition metal oxides coupled with nitrogen-doped graphene sheets could be used as noble-metal-free substitute catalysts for the ORR with comparable efficiency, owing to the unique charge transfer at the graphene-metal interface (Guo et al., 2012) and the synergistic effects between nitrogen, carbon, and metal atoms (Liang et al., 2011, 2013; Liang Y. et al., 2012; Wu et al., 2012; Zhang et al., 2013; Mao et al., 2014). The most commonly used support, however, chemically converted graphene, (Hummers and Offeman, 1958; Liang et al., 2011; Guo et al., 2012; Liang J. et al., 2012; Zhang et al., 2013; Chen et al., 2014; Mao et al., 2014) is vulnerable to stacking and aggregation during the reduction processes, which would decrease the specific surface area and hamper mass transfer, thereby compromising the overall properties of the electrocatalyst (Wen et al., 2012; Wu et al., 2012). In addition, the transition metal oxides are not electrically conductive, which would increase the electrical resistance and inhibit the charge transfer between active catalysts and current collectors (substrates), thereby limiting the catalytic performance of the electrocatalyst under identical conditions (Liang Y. et al., 2012; Zheng et al., 2014). In the light of these issues, exploring simple approaches to produce a high-specific-surface-area nitrogen-doped graphene support and to minimize the resistance between the transition metal oxide particles has become necessary for further improving the electrocatalytic performance of this type of electrocatalyst.

We herein report the facile and surfactant-free synthesis of novel cobalt/cobalt oxide core-shell nanostructures supported on nitrogen doped reduced graphene oxide aerogel (denoted as Co/CoO-NGA) and explore this material as an ORR electrocatalyst. The replacement of the cores of metal oxide particles with pure metal would greatly increase electric conductivity and facilitate electron transfer to and from the nanoparticle (Guo et al., 2012; Zhuang et al., 2014). In contrast to the traditional complex wet-chemistry method for controlling the growth of shells on pre-synthesized metal nanoparticles, (Luo et al., 2008; Guo et al., 2012; Zhuang et al., 2014) our synthetic procedure provides a simple and economically feasible method for large-scale synthesis of core-shell nanostructures. In addition, the designed three-dimensional (3D) structures in nitrogen-doped reduced graphene oxide aerogel (denoted as NGA) could effectively protect the flat sheets from significant aggregation, offering more active sites and multiple mass transport pathways for the ORR (Xu et al., 2010; Wu et al., 2012; Hu et al., 2013). As a result, the electrocatalyst shows comparable ORR catalytic activity to commercial Pt/C catalyst (20 wt. % Pt on Vulcan XC-72, E-Tek), but with much better stability and excellent methanol tolerance. More importantly, we at last conduct single anion exchange membrane fuel cell (AEMFC) test, which evidences the exceptional catalytic ORR performance of the Co/CoO-NGA under practical environment and also provides further confidence in developing low-cost cobalt metal oxides-nitrogen doped carbon based electrocatalysts for the AEMFC. To our best knowledge, some of cobalt oxides and graphene aerogel composites were reported, (Yuan et al., 2013; Zhang et al., 2014; Zheng et al., 2014) however the core-shell structured cobalt/cobalt oxides and the doping of nitrogen into graphene aerogel were not mentioned in these literatures.

## Experimental

### Materials

Graphite (325 mesh, Sigma), Sulphuric acid (H_2_SO_4_, concentrated, Ajax Finechem), phosphoric acid (70%, H_3_PO_4_, Ajax Finechem), Potassium permanganate (KMnO_4_, Sigma), hydrogen peroxide (H_2_O_2_, Sigma), Hydrochloric Acid (33%, HCl, Ajax Finechem), Cobalt Nitrate Hexahydrate (Co(NO_3_)_2_ 6H_2_O), Urea (Sigma).

### Synthesis

Graphene oxide (GO) was prepared from natural graphite flakes (Sigma) using a modified Hummers method, which was described elsewhere (Hummers and Offeman, 1958; Marcano et al., 2010). In a typical synthesis, 80.7 mg Co(NO)_3_·6H_2_O, 1 g urea and 15 ml of 2 mg mL^−1^ well-dispersed GO were mixed for 10 min before putting into an autoclave at 170°C for 15 h. After the reaction, the hydrogel was carefully taken out with a tweezers and directly freeze-dried for 12 h. After freeze-drying the product was annealed at 800°C for 2 h to produce Co/CoO-NGA under argon. In this study, the annealing temperature temperatures was finally set to 800°C, because no observed changes were seen on the XRD pattern while the nitrogen doping content would be decreased when increasing the temperature above 800°C.

To synthesize core shell cobalt/cobalt oxide (Co/CoO) supported on 2 dimensional (2D) nitrogen reduced graphene oxide (rGO) sheets (denoted as Co/CoO-NG), the mixed solution were placed into a flask with refluxing at 170°C for 15 h with magnetic stirring and after the reaction, the solid products were obtained via evaporating and then thermal treated with the same manner above. To synthesize nitrogen doped reduced graphene oxide aerogel (NGA), the synthetic procedure is the same as above except no cobalt salts were added before the hydrothermal process. To synthesize cobalt oxide on NGA (CoO-NGA), the procedures were the same except the temperature was set at 400°C for 2 h.

### Physical characterization

Scanning electron microscopy (SEM) images were obtained using a JEOL-7500FA. TEM images, High resolution (HR)-transimission electron microscopy (TEM) images were collected with a JEOL JEM-2100F transmission electron microscope (TEM) operated at 80 kV. Scanning transmission electron microscopy (STEM) images and Energy dispersive X-Ray spectrum (EDS) mapping analysis were obtained with a JEOL JEM-ARM200F operated at 80 kV. Samples for TEM, EDS, STEM were prepared by dropping one drop of the catalysts ethanol dispersion on a holy carbon film coated copper grid (200 mesh). Ethanol dispersion was prepared from sonication using a probe sonicator (Brandson S-250D) operated at 50% aptitude for 1 h. The The powder X-ray diffraction (XRD) patterns were collected using a GMC MMA X-ray powder diffractometer with Cu Kα radiation (λ = 1.5418 Å). X-ray photoelectron spectroscopy (XPS) spectra were collected using a Thermo Scientific K-Alpha instrument.

### Electrochemical characterization

The rotating-disk electrode (TF-RDE) technique were employed to evaluate the electrochemical performance, as reported elsewhere (Liang et al., 2011; Liang J. et al., 2012; Chen et al., 2014). Typically, the electrocatalysts were dispersed in Nafion/H_2_O/isopropanol (m/m/m = 0.05/10/50) to obtain a electrocatalysts ink (2 mg ml^−1^) via a probe sonicator (Brandson S-250D). The thin catalyst film/layer was fabricated through dropping 60 μL of the catalyst ink on a glassy carbon (GC) rotating disk electrode (RDE, 0.2457 cm^2^, Pine Research Instrumentation). The RDE testing system, a standard three-electrode cell with a Pt-wire counter electrode and a KNO_3_(aq) saturated (10 wt.%) Ag/AgCl reference, was then connected to a CHI 720c bipotentiostat (CH Instruments) for the evaluation of electrochemical performance.

Typically, the linear sweep voltammetry (LSV) technique was applied to investigate the ORR polarization curves in both nitrogen and oxygen saturated electrolytes sweeping from 0.1 to −0.8 V at a scan rate of 10 mV s^−1^ with various rotation speeds (100, 400, 900, and 1600 rpm). Koutecky-Levich (K-L) plots, to determine the the number of electron involved, could be drafted from the obtained ORR polarization curves at different potentials. The number of electrons involved per O_2_-molecule reduction can be determined using the Koutecky-Levich (K-L) equation (Equations 1, 2).

(1) $$\begin{array}{l} j=1/j_{k}+1/B\omega^{0.5} \end{array}$$

(2) $$\begin{array}{l} \left[B=0.2n\text{F}{\left(D_{O2}\right)}^{2/3}{\left(v\right)}^{-1/6}C_{O2}\right] \end{array}$$

Where, *j*_*k*_ is the kinetic current, ω is the electrode rotation rate, *n* is the transferred electron number, *F* is the Faraday constant (*F* = 96485 C mol^−1^), *D*_*O*2_ is the diffusion coefficient of O_2_ = 1.9 × 10^−5^ cm^2^ s^−1^, υ is the kinetic viscosity (0.01 cm^2^ s^−1^), and *C*_*O*2_ is the bulk concentration of O_2_ (1.2 × 10^−6^ mol cm^−3^).

Methanol tolerant was carried out through a chronoamperometry technique at −0.3 V and with a rotation speed of 1600 rpm with subsequently introducing of oxygen and methanol (1 M) at set time. Stability tests were carried out under identical conditions in an oxygen-saturated electrolyte for up to 13000 s.

### Anion exchange membrane single fuel cell test

The membrane used for AEM fuel cell is type of S80 (University of Surrey; Varcoe and Slade, 2006) with thickness of 80 μm and ion exchange capacity (IEC) around 1.3 meq g^−1^. The electrodes (both anode and cathode) were prepared as described in our previous report (Varcoe and Slade, 2006). In brief, the electrocatalyst ink, with 15 wt.% poly(vinybenzyl chloride) dissolved in ethyl acetate, were directly sprayed onto the gas diffusion layers (GDLs, 5 cm^2^) with a control loading level of 0.4 mg catalysts per cm^2^ and were subsequently immersed in undiluted *N, N, N*′, *N*′-tetramethylhexane-1,6-diamine (TMEDA) for 24 h and then washed thoroughly with water. The Pt/C was used as the electrocatalysts for anodes at a loading level of 0.2 mg_Pt_ cm^−2^. For comparison purpose, the commercial E-Tek Pt/C with Pt loading about 20 wt.% and the Co/CoO-NGA were chosen and used as catalysts for cathodes. Before single fuel cell testing, the AAEMs and GDL electrodes were submeged into KOH(aq) (1 M) solution for 1 h to give alkaline anion-exchange materials (OH^−^ conducting polymer electrolyte and cross-linked ionomer). The membrane electrode assembly (MEA) was prepared by sandwiching the anode and cathode GDLs. Then, the resulted AAEM was investigated using a standard 850e fuel cell testing system (Fuel Cell Technologies, Inc., USA) operated under the humidified hydrogen and oxygen (RH = 100%) at temperature of 50°C without back pressure, with a controlled gas flow of 0.20 L·min^−1^. The steady-state polarization curves were recorded using a current scan method by holding the testing cell at each point for 60 s with a scan rate of 10 dec/pt from 0 A to about 2 A.

## Results and discussions

**Figure 1:**
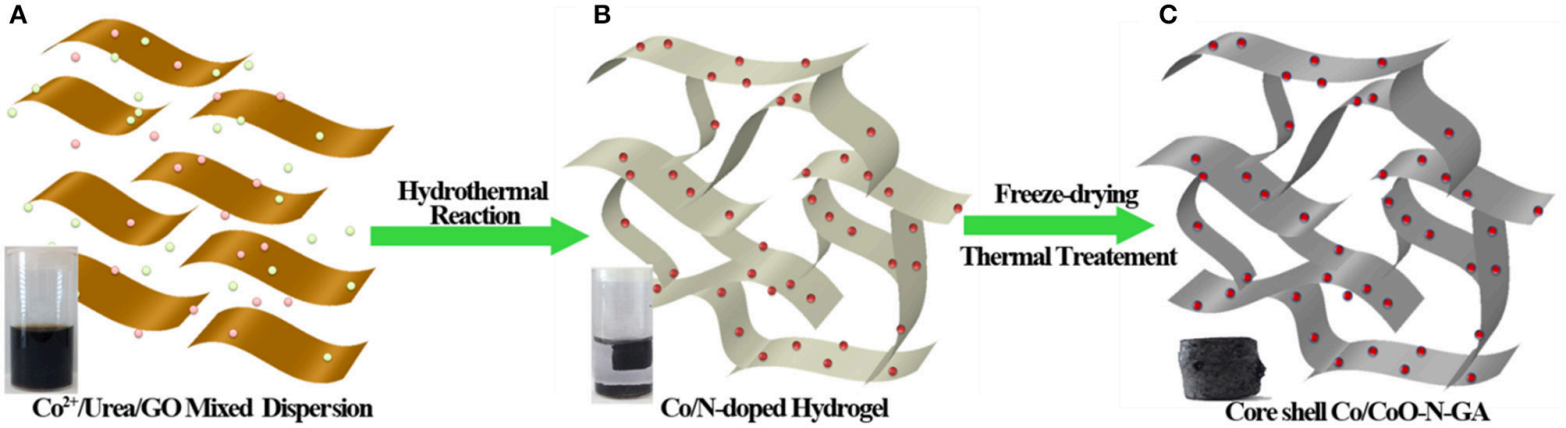
**Schematic illustration of the fabrication process for Co/CoO-NGA based on a hydrothermal method**. **(A)** Dispersion of Co^2+^/urea/GO; **(B)** Co/N-doped rGO Hydrogel; **(C)** Core-shell Co/CoO-N-GA after thermal treatment.

The fabrication process for Co/CoO-NGA is illustrated in Figure 1. In a typical synthesis, GO, cobalt salts, and urea are first dispersed in water (Figure 1A) and then hydrothermally assembled to produce a graphene-based 3D hydrogel (Figure 1B). In this step, the cobalt-hydroxide-carbonate intermediates are grown on rGO sheets, and nitrogen species will simultaneously be incorporated into the rGO lattice (Su et al., 2013). The hydrogel was then freeze-dried to maintain the 3D structure and annealed at 800°C under argon. In this way, nitrogen atoms in the graphene lattice are rearranged at high temperature, and the cobalt-hydroxide-carbonate is eventually converted into metallic cobalt. After exposure to air, the surface of the Co is oxidized, forming the Co/CoO core-shell nanostructures (Figure 1C). For comparison, core shell cobalt/cobalt oxide supported on 2 dimensional (2D) nitrogen-doped reduced graphene oxide sheets (Co/CoO-NG), nitrogen doped reduced graphene oxide aerogel (NGA), and CoO supported on nitrogen-doped reduced graphene oxide aerogel (CoO-NGA) were also synthesized (See Section Experimental for details).

**Figure 2:**
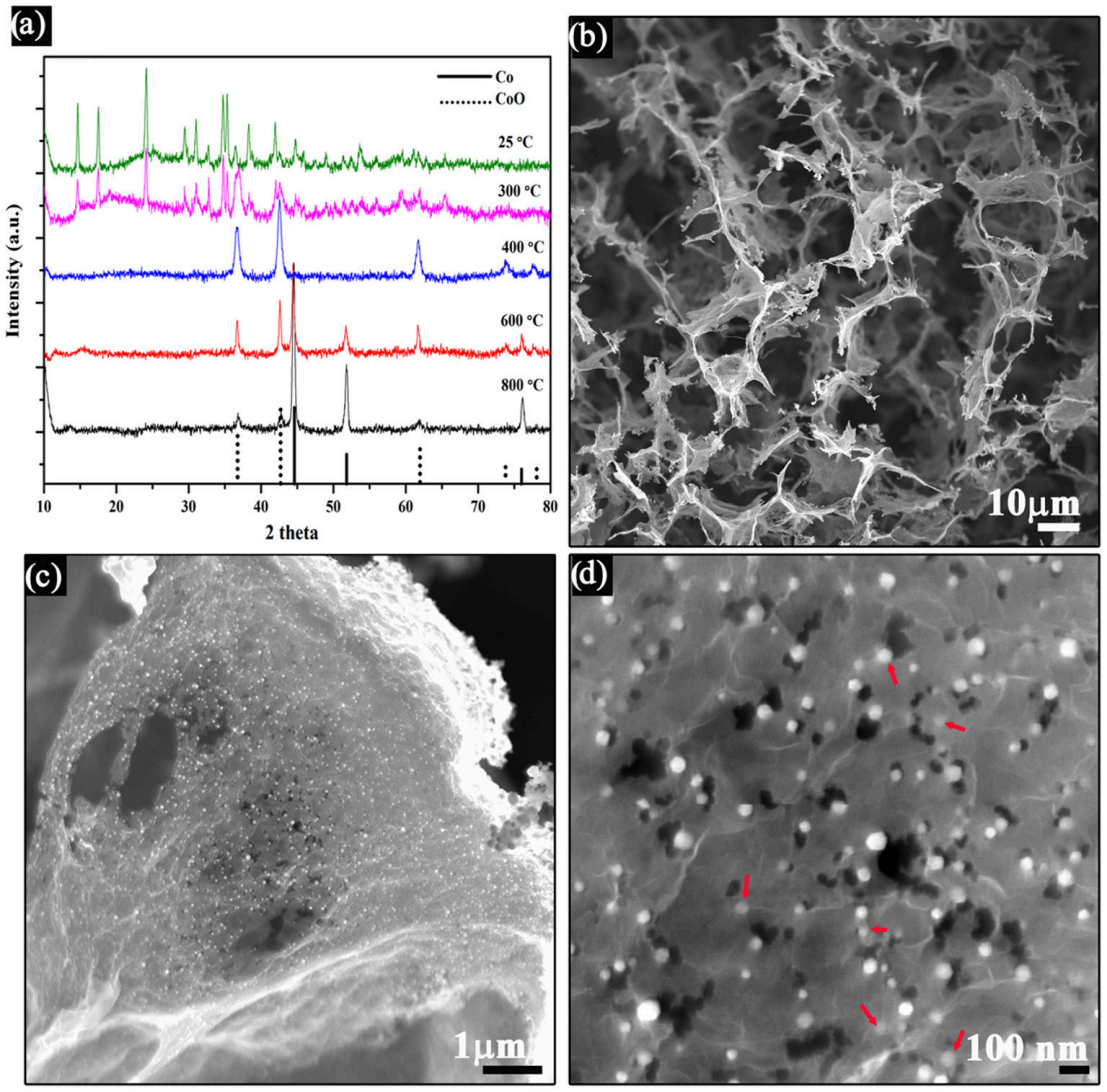
**(A)** XRD patterns of the synthesized products annealed with different temperature, **(B–D)** SEM images of the Co/CoO-NGA obtained at annealing temperature of 800°C. Red arrow shows the Co/CoO nanoparticles on graphene.

The synthesized product was first examined using X-ray powder diffraction (XRD, Figure 2A), showing the coexistence of the metallic cobalt and cobalt oxide in the final products, suggesting that the cobalt intermediates could indeed be reduced to metallic cobalt. After the hydrothermal process, cobalt-hydroxide-carbonate complex was obtained (Guo et al., 2013). No virtual crystal changes could be observed when the annealing temperature was below 300°C, indicating the cobalt-hydroxide-carbonate was thermal stabile below 300°C. Cobalt (II) oxides could be obtained when the annealing temperature was set to 400°C, indicating the decompositon of the cobalt-hydroxide-carbonate, as reported by many other literatures (Liang et al., 2013; Mao et al., 2014). Further increase of the annealing temperature could lead the formation of metallic cobalt. In this study, the temperatures was finally set to 800°C to completely reduce the Co^2+^, because no observed changes were seen on the XRD pattern while the nitrogen doping content would be decreased when increasing the temperature above 800°C. Besides, it is worthwhile to note even metallic cobalt could not be formed in the ammonium reducing environment at 800°C, (Liang Y. et al., 2012) the successful synthesis of cobalt metallic in this experiment could be ascribed to the presence of urea in the cobalt-hydroxide-carbonate complex, which would facilitate the crystallization of cobalt metal by producing a regional reducing environment when decomposing at high temperatures (Querejeta-Fernández et al., 2010).

Scanning electron microscopy (SEM) was used to study the structure and morphologies of the products. A 3D interconnected macroporous rGO structure can be clearly discerned in the SEM images, indicating that the integration of 3D graphene could effectively inhibit the graphene sheets from stacking (Figure 2B). Noticeably, there was a homogeneous decoration of metal oxide nanoparticles on both sides of the graphene sheets, with particle size from 20 to 50 nm (Figure 2C). Some particles were even encapsulated within the graphene layer structure (Figure 2D), which would suppress the dissolution of the nanoparticles during electrochemical processes and thereby improve the stability of the electrocatalyst (Wu et al., 2012).

**Figure 3:**
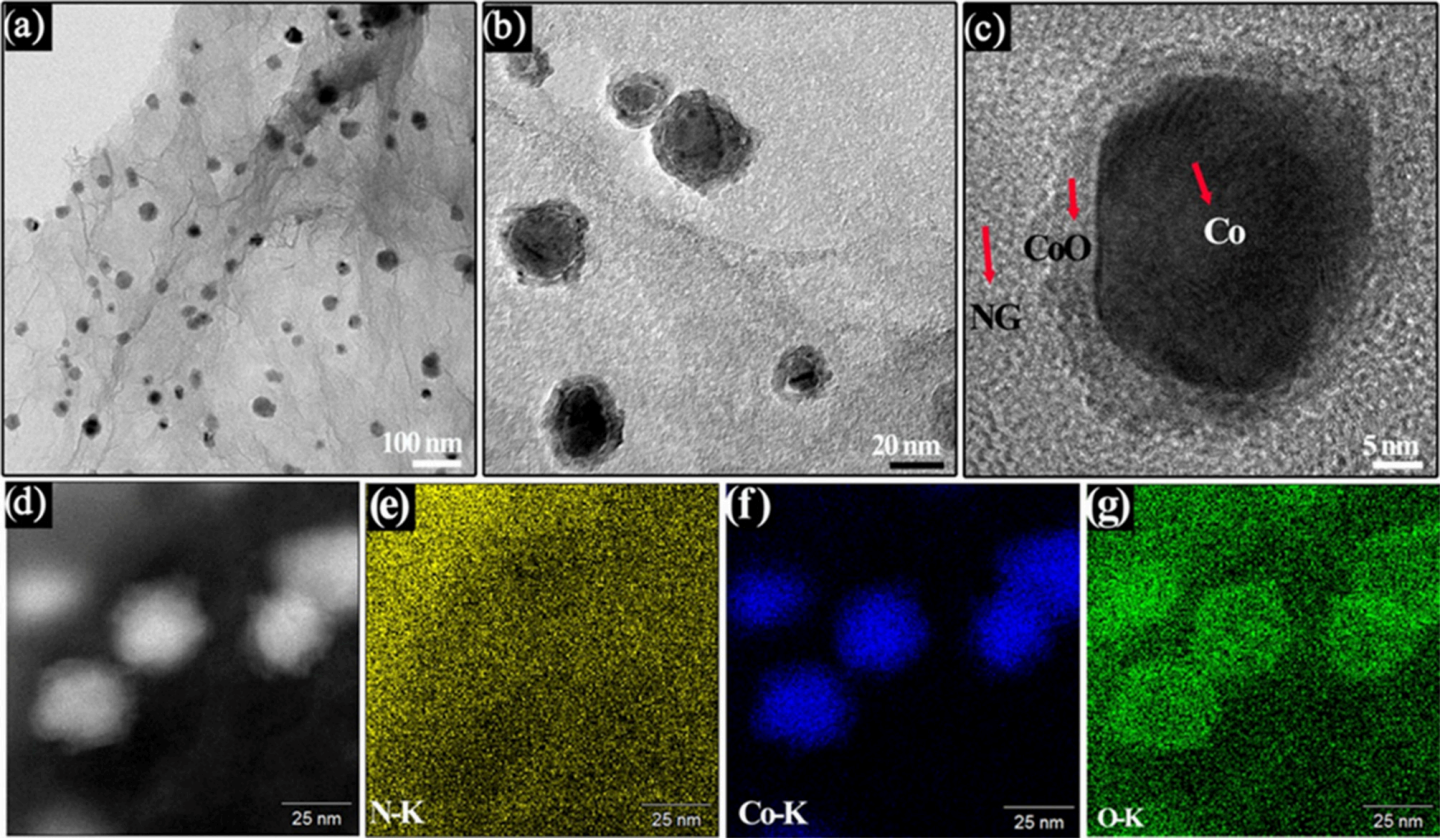
**(A,B)** TEM images, **(C)** HR-TEM images and **(D)** STEM images of the Co/CoO-NGA, **(E,F)** EDS element mapping profile of the Co/CoO-NGA, **(E)** N, **(F)** Co, and **(G)** O.

To gain insight into the structural information of the Co/CoO-NGA, the TEM analysis was conducted on the Co/CoO-NGA, also confirming the homogeneous decoration of cobalt nanoparticles with size around 20–30 nm supported on the porous graphene structure (Figure 3A). In addition, distinct differences on cores and shells of the nanoparticles were observed on high-magnification TEM images (Figure 3B), confirming the formation of core-shell structure which was then investigated in detail by high resolution TEM (HRTEM, Figure 3C). It can be seen that the shell and core are both well-crystallized, and the three types of subunits, NGA, CoO, and Co, are highly integrated, suggesting strong bonding between them, which would enhance the interaction between the metal oxide nanoparticles and the graphene support, promoting electron and charge transfer among the active sites and thus increasing the electrocatalytic performance.

Scanning transmission electron microscopy (STEM) with energy dispersive X-ray spectroscopy (EDS) mapping analysis was employed in order to determine the element distribution in the samples. The STEM images also revealed structural differences in the shell and core of the nanoparticles (Figure 3D). The element mapping evidences uniform nitrogen doping in the products, suggesting the effectiveness of this method in achieving uniform doping of graphene (Figure 3E). Moreover, it can be seen that the intensity for oxygen is much higher around the shells of the cobalt nanoparticles, which is attributed to the CoO of the shell and further confirms the successful synthesis of a Co/CoO core-shell nanostructure (Figures 3F,G).

**Figure 4:**
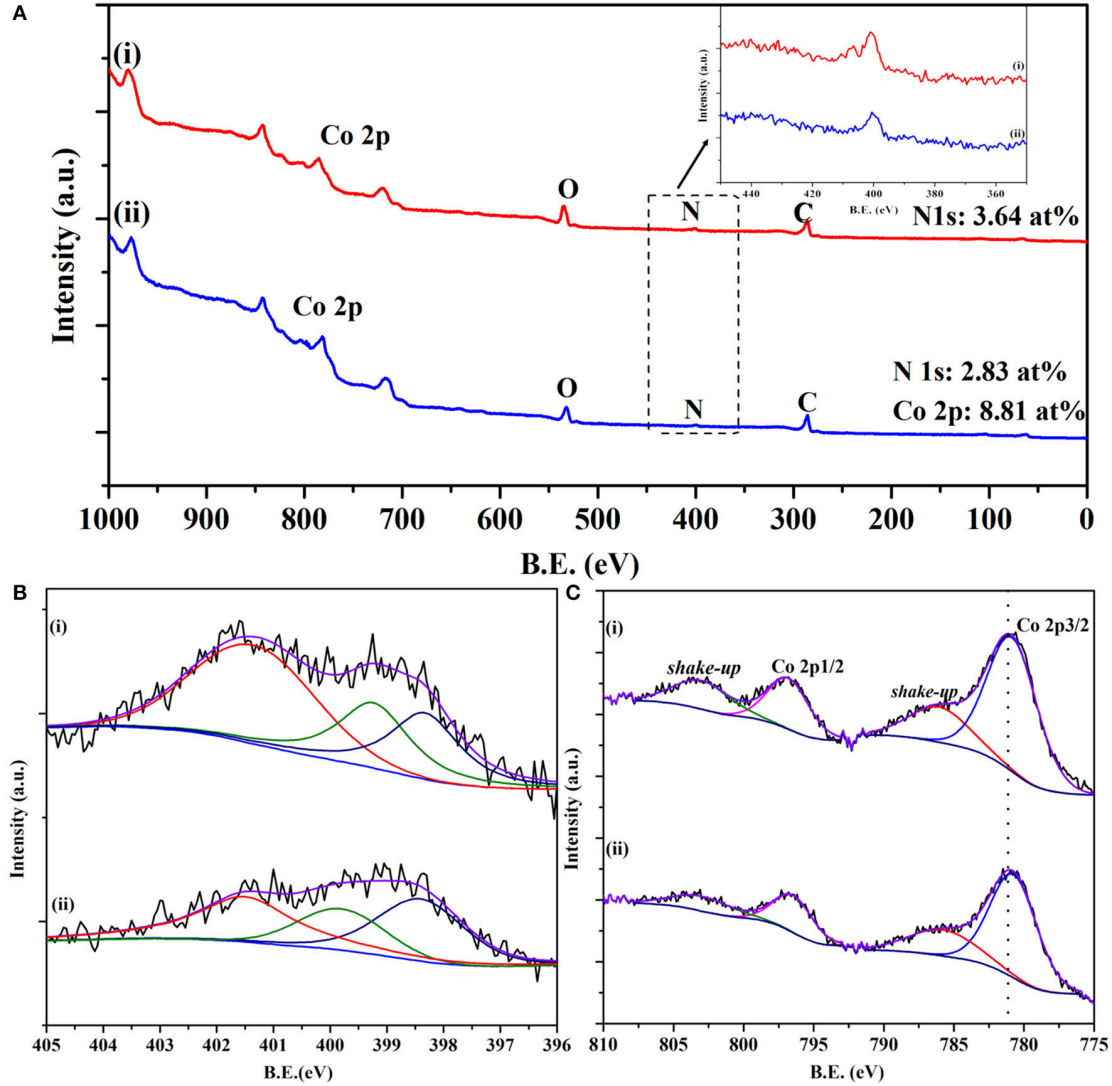
**(A)** XPS surveys of the synthesized products, **(B)** high-resolution N1s scan, and **(C)** high-resolution Co 2p scan of (i) Co/CoO-NGA, (ii) CoO-NGA.

X-ray photoelectron spectroscopy (XPS) was also employed to investigate the chemical states of elements present in the electrocatalysts. A nitrogen content of 2.83 at% was detected for the Co/CoO-NGA, and the cobalt content is 8.81 at% (Figure 4A). To further investigate the nitrogen configuration, high resolution XPS spectra for N 1s were obtained and could be fitted to three peaks: pyridinic N (398.5 eV), pyrrolic N (399.8 eV), and quaternary N (401.5 eV), (Wang et al., 2010) as shown in Figure 4B. It could also be seen that more quaternary N and pyridinic N were detected in the spectrum, which would be beneficial for the ORR as previous studies showed that the quaternary and pyridinic N are more active in catalyzing oxygen than the pyrrolic N (Yang et al., 2012). The high resolution XPS spectrum for Co 2p was also obtained and fitted in order to study the electronic state of Co (Figure 4C). It can be seen that the Co 2p spectrum is spin-orbit split into 2p_1/2_ and 2p_3/2_ components. Broad peaks around 786.3 eV were found in the Co 2p_3/2_, which could be ascribed to the shake-up satellite of the cobalt ions, indicating that the metal oxide is cobalt(II) oxide rather than other cobalt oxides (Yang et al., 2009). In addition, as XPS is a surface sensitive detective technique, it is reasonable to infer that the absence of the metallic cobalt peak in the XPS reveals that all Co(0) centers are covered by the oxide layer.

**Figure 5:**
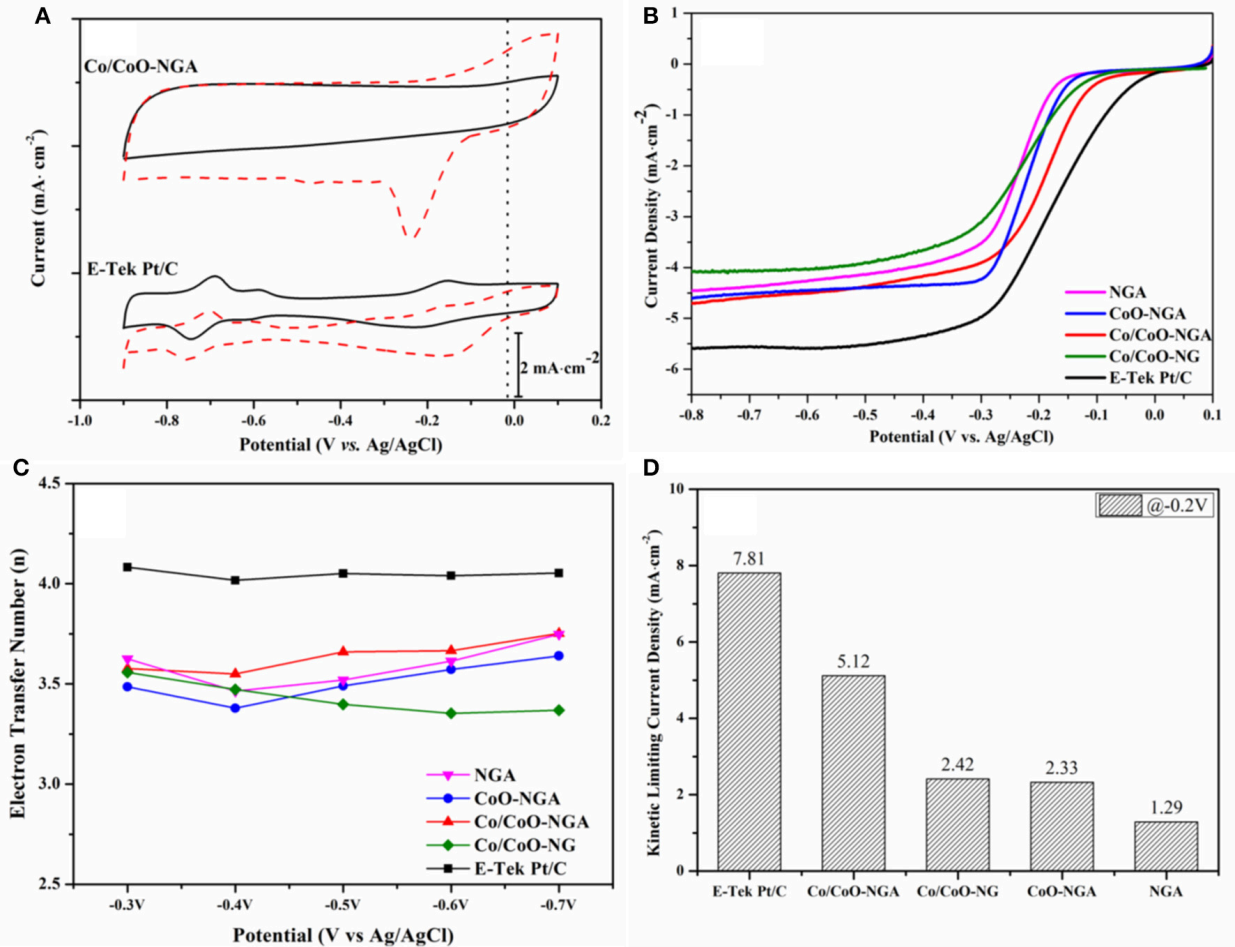
**Electrochemical performances of the Co/CoO-NGA, Co/CoO-NG, CoO-NGA, NGA, and E-Tek Pt/C electrocatalysts: (A) CV curves of the Co/CoO-NGA and the Pt/C in N_2_ (black lines) and O_2_ (red dashed lines) saturated electrolyte (50 mV·s-1); (B) LSV curves of the electrocatalysts in O_2_ saturated electrolyte at 1600 rpm; (C) electron transfer number of the electrocatalysts at different potentials from −0.3 to −0.7 V (vs. Ag/AgCl); and (D) kinetic limiting current density of the electrocatalysts at −0.2 V (vs. Ag/AgCl)**. The electrolyte is KOH (aq, 0.1 mol dm^−3^).

Cyclic voltammetry (CV) curves were obtained and studied the ORR performance of the synthesized electrocatalysts. As shown in Figure 5A, a quasi-rectangular featureless voltammetric current trace within the potential range of −0.9 to 0.1 V was observed for the Co/CoO-NGA in the N_2_-saturated solution, this being a result of the typical capacitance influence on porous carbon materials (Liang J. et al., 2012; Parvez et al., 2012). In contrast, when the electrolyte was saturated with O_2_, a well-defined ORR peak centered at −0.25 V was detected for the Co/CoO-NGA. The onset potential (at which oxygen began to be reduced) was at ~ −0.06 V for the Co/CoO-NGA, which was comparable to that of the Pt/C (−0.01 V) suggesting that the core-shell structured Co/CoO-NGA had the quality high-level electrocatalytic performance relative to the high cost Pt/C electrocatalysts.

To gain insight into the ORR activities and kinetics of the various electrocatalysts, the steady state ORR polarization curves were obtained using linear sweep voltammetry (LSV) at a sweep rate of 10 mV s^−1^ (Figure 5B). To compare the catalysts' performances, the half-wave potential (*E*_1/2_), at which the current is a half of the limiting current, was calculated. For the Co/CoO-NGA, the *E*_1/2_ is −0.189 V, which is very close to that for Pt/C (−0.170 V) and much higher than that for Co/CoO-NG (−0.236 V), suggesting the 3D macroporous graphene support could provide more mass and ion transfer channels and more active sites for the ORR compared with the plane graphene sheets thereby increasing the electrocatalytic activities. While compared with the *E*_1/2_ for CoO-NGA (−0.219 V), the relatively high *E*_1/2_ for the Co/CoO-NGA reveals the unique core shell structure of cobalt and cobalt oxide could largely improve the electrocatalytic ORR performance, which might be due to the increased electron transfer rate and the decreased electrical resistance of the electrocatalysts.

To study the kinetics of the catalytic ORR, the steady state ORR polarization curves were also collected at various rotation speeds, and the corresponding Koutecky-Levich (K-L) plots were determined from the related ORR polarization curves at different potentials. The electron-transfer numbers (*n*) were thereafter calculated at various potentials and are shown in Figure 5C. Generally increasing *n*-values were seen as the potential became more negative; for Co/CoO-NGA, the *n*-value is always above 3.5 and higher than for Co/CoO-NG, CoO-NGA, and NGA, suggesting a greater proportion of 4-electron reduction of oxygen than with the other catalysts. The kinetic limiting current density (*j*_*k*_) was also calculated based on the K-L equation at −0.2 V (vs. Ag/AgCl), and shows a similar trend to the other comparisons (Figure 5D, See Section Experimental for detailed calculations). At −0.2 V, the *j*_*k*_ for Co/CoO-NGA is 5.1 mA·cm^−2^, which is significantly higher than those for Co/CoO-NG, CoO-NGA, and NGA. These comparisons clearly indicate the improved electrocatalytic performance of the core-shell porous Co/CoO-NGA electrocatalyst relative to the Co/CoO-NG, CoO-NGA, and NGA electrocatalysts; this could be due to well-defined 3D porous structure of nitrogen doped graphene supports, as well as the accelerated electron transfer in the core-shell structure as compared to the pure metal oxide phase and the 2D nitrogen doped grapheme (Liang et al., 2011, 2013; Liang Y. et al., 2012; Wu et al., 2012; Zhang et al., 2013; Mao et al., 2014).

**Figure 6:**
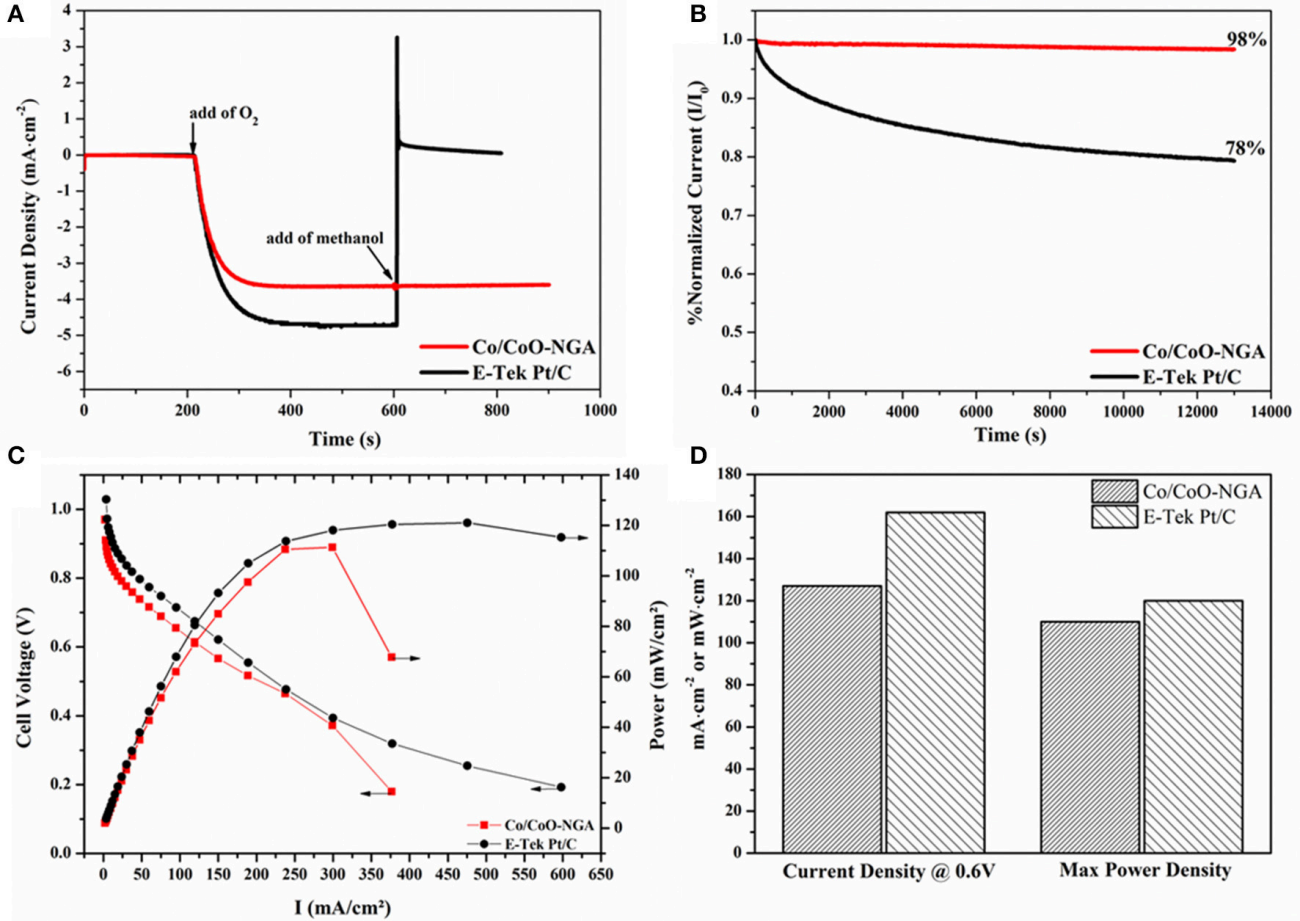
**Chronoamperometric responses at −0.3 V (vs. Ag/AgCl) of the Co/CoO-NGA and E-Tek Pt/C at 1600 rpm: (A) with the addition of oxygen and methanol (1 mol dm-3), (B) in pure O_2_-saturated electrolyte up to 13000 s. (C)** Polarization curves of AEMFC single cells containing Co/CoO-NGA and E-Tek Pt/C as cathode electrocatalysts, respectively. **(D)** Comparisons of current density at 0.6 V and maximum power density derived from the single cell tests in **(C)**.

The selectivity toward methanol of the catalyst is a key factor in real application in fuel cells, because the relatively small fuel molecules (i.e., methanol) can cross over from the anode through the membrane and react with the catalyst in the cathode, causing poor ORR performance and further reducing the cell efficiency (Liu et al., 2013). To this end, the selectivity of Co/CoO-NGA and Pt/C were compared through chronoamperometric measurements with subsequent introduction of oxygen and methanol, as displayed in Figure 6A. The introduction of oxygen led to a significant increase in the current density, and a stable ORR current was reached after several minutes for both catalysts. After the addition of methanol, however, a distinct decrease in current was observed for the Pt/C catalyst, indicating that methanol oxidation had occurred at the cathode, i.e., the selectivity of the Pt/C was poor. In contrast, for Co/CoO-NGA, the current remained almost unchanged after the addition of methanol, reflecting its superior selectivity, and methanol tolerance.

The stability of the Co/CoO-NGA was also assessed and compared to Pt/C by the chronoamperometric technique (Figure 6B). In longer term tests (up to 13000 s), only a 2% current loss was observed with Co/CoO-NGA, whereas for commercial Pt/C, the current loss was about 22%; this clearly indicated that the Co/CoO-NGA has much better stability than commercial Pt/C in this test, revealing its superiority as a long-term stable catalyst in practical operation. These comparisons between Co/CoO-NGA and Pt/C on methanol tolerance and selectivity (Figures 6A,B) suggest that Co/CoO-NGA could be a better electrocatalyst than commercial Pt/C for application in direct methanol fuel cells (DMFCs).

Anion exchange membrane fuel cell (AEMFC) testing was finally conducted in order to determine the practical performance of the Co/CoO-NGA in a real-world fuel cell application (Figure 6C). To our surprise, under identical testing conditions, the Co/CoO-NGA electrocatalyst shows similar catalytic behavior to commercial Pt/C. At the practicable operating potential (~ 0.6 V), the Co/CoO-NGA electrocatalyst delivered a current density of 127 mA·cm^−2^, 78% of that for Pt/C (162 mA·cm^−2^, Figure 6D). Additionally, the maximum power output with Co/CoO-NGA was 110 mW·cm^−2^, over 90% of that with Pt/C (120 mW·cm^−2^, Figure 6D). These comparisons clearly indicate that Co/CoO-NGA would have similar catalytic performance to commercial Pt/C in real-world applications, suggesting that it could work efficiently as a low cost ORR electrocatalyst under practical operating conditions.

## Conclusion

In summary, we have demonstrated a facile method for fabricating core-shell cobalt/cobalt oxide nanostructures using 3D N-doped reduced graphene oxide as the supporting material (Co/CoO-NGA) without adding any surfactants. Co/CoO-NGA was explored as an electrocatalyst toward the ORR in alkaline medium, where it showed excellent ORR catalytic performance, superior methanol tolerance, and high durability. It can be concluded that the improved electrocatalytic performance toward the ORR could be attributed to the significant electron and charge transfer properties of the synergistic enhancement from the nitrogen-metal-carbon interactions, the unique core-shell structures, and the 3D structure of the supporting material in the developed electrocatalyst. We believe that this synthetic method has the potential to be further extended to the synthesis of other related core-shell transition-metal nanostructures, and would have benefits in the design and development of economically feasible, low-cost, and environmentally friendly electrocatalysts for the next-generation of alkaline fuel cells.

## Author contributions

MW conducted the most experiments as part of his Ph.D. project. JC and JW conceived and designed the project as MW's Ph.D. supervisor. MW, YH, DW, and DS performed the characterization and data analysis. All authors involved the analysis of experimental data and manuscript preparation.

### Conflict of interest statement

The authors declare that the research was conducted in the absence of any commercial or financial relationships that could be construed as a potential conflict of interest.
